# The complete chloroplast genome and phylogenetic analysis of *Cuminum cyminum*

**DOI:** 10.1080/23802359.2020.1722037

**Published:** 2020-02-07

**Authors:** Jing Zhou, Zhenwen Liu

**Affiliations:** a School of Pharmaceutical Science & Yunnan Key Laboratory of Pharmacology for Natural Products, Kunming Medical University, Kunming, China;; b Key Laboratory for Plant Diversity and Biogeography of East Asia, Kunming Institute of Botany, Chinese Academy of Sciences, Kunming, China

**Keywords:** *Cuminum cyminum*, complete chloroplast genome, phylogeny

## Abstract

*Cuminum cyminum* (Apiaceae) is an economically important plant, whose fruits are usually used as flavoring, and also have pharmacological activities such as antioxidant, antibacterial, hypolipidemic, and so on. In this study, we assembled and annotated complete chloroplast (cp) genome sequence of *C. cyminum*. The results showed that the complete cp genome of *C. cyminum* was 157,839 bp in length, consisting of a large single-copy (LSC) region of 83,927bp, a small single-copy (SSC) region of 17,598bp, and two inverted repeat regions (IRa and IRb) of 28,157bp. In total, 131 genes were annotated, comprising of 86 protein-coding genes, 37 tRNA genes, and 8 rRNA genes. The phylogenetic analysis indicated that *C. cyminum* belongs to the tribe Scandiceae, and showed close relationship with *Daucus carota*.

*Cuminum cyminum* L., an economically important plant belonging to the family Apiaceae, is indigenous to SW Asia and the Mediterranean region, but is today widely cultivated. Its aromatic fruits (cumin) are popular for its flavor, and has been used in Chinese and Ayurvedic medicine for the treatment of dyspepsia, diarrhea, and jaundice (Sheh et al. 2005; Srivsatava et al. 2011). Studies on this species have focused on describing its chemical constituents (Yan et al. 2002; Hashemi et al. 2009; Zhou et al. 2011) and pharmacological activity (Lee 2005; Sultana et al. 2010; Chen et al. 2011), rarely involved in its genomes. Here, the complete chloroplast (cp) genome sequence of *C. cyminum* is characterized, and its phylogenetic relationships with related taxa in Apiaceae are revealed.

The total genomic DNA was extracted from the leaves of *C. cyminum* collected from Kuerle (41°36′5.34″N, 86°03′25.66″E), Xinjiang of China using a Universal Genomic DNA Extraction kit (Tiangen Biotech, Beijing, China) following the manufacturer’s protocol. Voucher specimen was deposited in KUN (Kunming Institute of Botany, Chinese Academy of Sciences, ZJ1706). Then, the genome sequencing were performed with Illumina Hiseq 2500 (Majorbio, Shanghai, China) platform with pair-end (2 × 300) library. The raw data were filtered using Trimmomatic with default settings (Bolger et al. 2014). Then paired-end reads of clean data were assembled into circular contigs using GetOrganelle.py (Jin et al. 2018) with *Daucus carota* (No. NC_008325) as reference. Finally, the plastome was annotated by the Dual Organellar Genome Annotator (DOGMA; http://dogma.ccbb.utexas.edu/) (Wyman et al. 2004) and tRNAscan-SE (Lowe and Chan 2016) with manual adjustment using Geneious (Kearse et al. 2012), and the physical map was drawn by OGDRAW (Lohse et al. 2007).

The plastomes of *C. cyminum* (GenBank accession number: MN901636) show the typical properties of Apiaceae and most other eudicot plastid DNAs in its structural organization, gene content, and gene arrangement. The total length of the chloroplast genome was 157,389 bp, with 37.80% overall GC content. With typical quadripartite structure, a pair of IRs (inverted repeats) of 28,157 bp was separated by a small single-copy (SSC) region of 17,598 bp and a large single-copy (LSC) region of 83,927 bp. The cp genome contained 131 genes, including 86 protein-coding genes, 37 tRNA genes, and 8 rRNA genes. Among these, 18 genes were duplicated in the inverted repeat regions.

To investigate its phylogenetic placement, a total of 30 cp genome sequences of Apiaceae were downloaded from the NCBI database. All sequences were aligned using the MAFFT (Katoh and Standley 2013) webserver (http://mafft.cbrc.jp/alignment/server/), a maximum likelihood (ML) analysis was constructed using RAxML (Stamatakis 2014) with 1000 bootstrap replicates, and *Bupleurum boissieuanum* used as outgroup (NC_036017). The results showed that *C. cyminum* belongs to the tribe Scandiceae, and it was closely related to *Daucus carota* (Figure 1). Meanwhile, the phylogenetic relationship in Apiaceae was consistent with previous studies, and this will be beneficial to construct a more reasonable phylogeny of Apiaceae.

**Figure 1.**
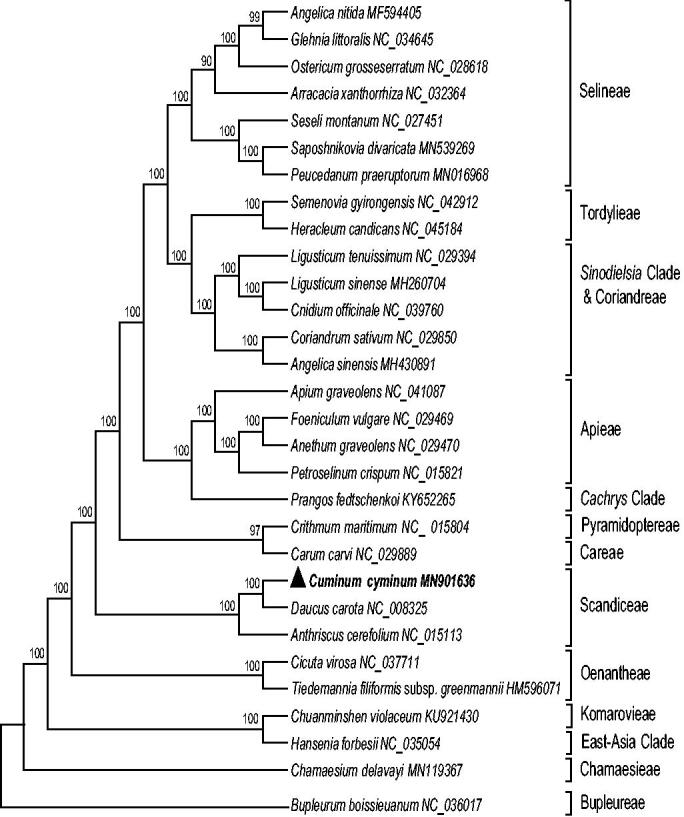
Maximum likelihood (ML) tree of 30 species in the family Apiaceae based on the complete chloroplast sequences using *Bupleurum boissieuanum* (NC_036017) as an outgroup. Numbers on the nodes are bootstrap values from 1000 replicates.
